# Supplementary material for: Racial and ethnic differentials in COVID-19-related job exposures by occupational standing in the US
Source: PLoS One. 2021 Sep 1;16(9):e0256085. doi: 10.1371/journal.pone.0256085 (PMC8409606; doi:10.1371/journal.pone.0256085)
Supplement: Attachment — Submitted filename: Response to Reviewers.docx [file pone.0256085.s003.docx]

We thank both reviewers for their helpful comments that we believe strengthened our paper. Below we present each of the comments (in italics) followed by our response.

**Reviewer #1**

- *The manuscript is very well written. The use of “occupational status” is innovative and informative. The manuscript has many strengths and few minor weaknesses. The findings provide more evidence on the racial disparities experienced by workers of color during COVID-19 pandemic for policy considerations.*

We thank the reviewer for this positive feedback.

- I just have a few suggestions:

*Typically, “occupational status” is defined by a combination of educational attainment and income. In this article, “occupational status” is defined by educational attainment only. Please comment on this point in your limitation section and whether your definition might or might not change your overall interpretation. Did you not have information on income?*

Duncan’s socioeconomic status index (SEI) does indeed combine occupational education and occupational status into a weighted index. However, Hauser and Warren (1997) – cited in the paper and below – argued persuasively that occupational education and occupational income should be used as two separate measures because they have different effects and much of the literature now does so. Our goal was to identify a simple proxy for the amount of power and control workers are likely to have in determining how they do their work and whether they have access to PPE and other workplace protections. We do have information on income, but we chose occupational education alone for this purpose to avoid complicating our results further by using two SES separate indices. Occupational education is easy to understand, frequently used in the sociological literature, and, compared to occupational income, less likely to have been altered by the pandemic. Furthermore, education is typically completed at young ages, whereas job choices (often based heavily on wage and salary rates) are made throughout the working years – so occupational income is more endogenous to the choice of job than is occupational education.

We have now changed the term we use from “occupational status” to “occupational standing” to avoid confusion.

We have revised our discussion of this issue in the text.

Reference: Hauser RM, Warren JR. (1997) Socioeconomic Indexes for Occupations: A Review, Update, and Critique: Sociological Methodology. Available: https://journals.sagepub.com/doi/10.1111/1467-9531.271028

- *The text in the figures are blurry and hard to read.*

We have completely revamped the figures. The figures now directly examine the overrepresentation vs. underrepresentation of frontline workers in high-risk occupations and show the results both numerically and by color (using a heatmap). These graphs are much easier to read and to interpret than the previous figures.

**Reviewer #2**

- *Language tweak throughout: use Black workers instead of Blacks*

Thanks for catching this. We have fixed this throughout the paper.

- *Introduction: since the submission of this paper, there have been a few key studies about occupational risks by race/ethnicity and by industry. Suggest updating the lit review*.

We have updated the literature review (primarily in the Introduction and Discussion sections), and now include studies published through March 2021 .

- *Introduction could be a lot meatier and get into some of the systemic drivers of potential reasons for greater exposure risk among low-wage and racial/ethnic minority workers. There’s been a ton of great work here—again, much of it published since this paper was submitted. But also the analysis needs more context and some data on, for example, disproportionate risks of hospitalizations and deaths by race and ethnicity, and hypothesized reasons.*

*It also needs more citation in general, for statements like that in lines 85-87.*

As noted above, we have revised the introduction to include new papers. We have also added language that refers to the “the long history and contemporary effects of structural racism on occupational segregation in the US” and provided references.

We agree that we could have written a much broader introduction discussing the role of structural racism and other social processes in producing the racial and ethnic distribution of workers by occupation, the reasons that lower wage workers are less likely to have access to PPE, and the disproportionate risks of hospitalizations and deaths. These are issues that we have written about at length in other work. However, this paper is specifically focused on the empirical evidence about the potential exposure to COVID-19 of frontline workers in different racial and ethnic groups. There has been extensive discussion in the press and academic publications about the issue, but limited empirical evidence – particularly evidence that includes proxy information on the likelihood of risk reduction mitigation, as we try to provide in this paper. We believe that it is essential to make these key empirical findings available to as broad an audience as possible as quickly as possible and, therefore, we have kept the narrow focus.

- *“Occupational status” is a very confusing term, and in reading the abstract I initially thought it was a typo. I assumed that it meant whether one was working or not working (synonymous with "employment status"), as it’s commonly used in that context in the occupational health literature. However, I see that you’re using it to mean “status” as in the social hierarchy, rather than status as in present/not present. I would suggest using a different term throughout.*

At your suggestion, we have changed the term to “occupational standing” throughout the paper, figures and appendices. “Occupational status” is a term from the sociological and social science literature used to refer to an individual’s position in the occupational hierarchy. However, we agree that it can be a confusing term for readers in other fields and so we have made the change.

- *In the intro, the authors appear to conflate race and ethnicity with “occupational status.” (lines 91-92). While the two often travel together, at this point in the manuscript they should be treated as separate unless they back up their assertions with the citations that appear later in the discussion.*

The lines referred to in the reviewer’s comment read:

91 Examining results by occupational status leads to a clearer picture of racial/ethnic differentials in

92 potential exposure to COVID-19 transmission.

Note that “occupational status” originally used in line 91 is now “occupational standing.” Our point was that differences in *the distribution of occupations* by race and ethnicity are likely to contribute to differences in exposure to COVID-19, since Black, Latino, and some other groups of workers are more likely to work in jobs without adequate protection compared to whites and some other groups. We are not in any way conflating race and ethnicity with occupational (or socioeconomic) status. The sentence in lines 91-92 was proceeded by the following text, which we believe makes the meaning of the text in 91-92 clear:

“In contrast, higher status workers have better access to risk mitigation measures such as personal protective equipment (PPE), frequent sanitation, enforced distancing, partitions, better ventilation, and new filtration systems.”

- *Does the ACS include both occupation and industry for individual workers? When linking with o*net, do you consider industry, or just occupation? Industry could be quite important for establishing baseline level of risk by occupational setting. For example, a janitor in a hospital would be much more highly exposed than a janitor in a warehouse*.

We agree that use of PPE and other forms of protection are likely to vary by industry as well as occupation. The ACS data contains both variables. However, the O*NET data provides exposures only for occupations (see <https://www.onetcenter.org/overview.html>). Thus, even if we subdivide ACS respondents both by occupation and industry, the exposure information we obtain from O*NET would be identical for respondents in the same occupation, regardless of industry.

- *For “frontline status,” how do you account for the differential job loss that industries and occupations experienced during the pandemic? Yes, a server in a restaurant would be considered frontline because it can’t be done from home, but almost all servers lost their jobs—reducing their exposure status in the process. Not accounting for potential job loss could lead to substantial exposure misclassification*

We agree that this is a potential problem, in our analysis and in all those which have sought to use pre-pandemic data to examine potential effects of the pandemic. The issue is even more complicated because “job loss” for individual workers changed a great deal during the pandemic depending on what was and was not open. In many states, some businesses were closed, then reopened for a while, then closed again, then reopened, etc. Many of these business that were unable to keep workers on the payroll while closed (although the Payroll Protection Program initially helped a lot of employers continue to pay workers) reemployed them when they reopened. The detailed data to take this issue into account is not yet available. However, we added a sentence to the text that refers to differences by race and ethnicity in job losses during the pandemic, and we specifically acknowledge our inability to account for job losses and unemployment.

With regard to the broader point about exposure misclassification, throughout the text we are now careful not to imply that these measures correspond to an individual’s experienced risk during the pandemic; rather, they correspond to racial and ethnic patterns of employment in high-risk occupations.

- *Table 1: suggest ordering by either size of the population or % frontline (for men) to make it easier to find the relevant information*

We believe that it is important to keep the order of racial/ethnic groups consistent with the graphs (and appendix tables) as well as to have the groups that have received the most attention listed first (White, Black and Latino) and to keep the four Asian groups together. We have changed the placement of Native Americans to come after the four Asian groups and directly before Asian and Pacific Islanders. This ordering is now consistent in Table 1, the figures, and the appendices.

- *The figures are nearly impossible to read, much less interpret. I printed out the manuscript and was unable to see the differences between the groups. In addition, readers are not good at comparing the relative size of bars on a figure aside from the categories at the beginning and the end, and it requires a heavy cognitive lift on the part of the reader. Strongly consider re-imagining how this data could be better visualized; I suggest any of Stephanie Evergreen’s books.*

As we noted in our response to Reviewer 1, we have completely redone the figures. The reader no longer is asked to assess the relative size of bars (an admittedly difficult and unreasonable task); these relative measures are computed directly and are shown as measures of overrepresentation and underrepresentation in various categories of occupations. Interpretation is aided by the use of a heatmap and a detailed numerical example.

- *The point you make in lines 276-277 is precisely why industry, in addition to occupation, is crucial to consider. While educational attainment is also important, industry is available at an individual level and so there is lower risk of misclassification bias. From the discussion I understand the reasons that you chose to rely on occupation rather than industry (murky definitions of essential workers), but could you not use the combination of industry/occupation for the exposure assessment, and only industry for the comparison?*

The lines referenced (276-277) have been rewritten to better clarify our focus on occupational standing and avoid comparing across industry. We now provide examples of two occupations within the healthcare sector—home health aides and physicians—to emphasize how occupations with similar levels of risk factors according to O*NET can have very different statuses. We have also included some citations to recent work to support this claim that lower status healthcare workers have received inadequate training in infectious disease protocol and faced severe shortages of personal protective equipment.

As noted above, only occupation-related exposure information is available from O*NET so we are unable to directly consider how these risk factors vary across industry.

- *Re: the limitation of women being forced out of the labor market due to having young children, I imagine that the ACS measures both child age and marital status. How could the authors leverage this information to do a sensitivity analysis?*

An analysis of women who had to leave the labor market to take care of children when schools were closed is very important, but is beyond the scope of this paper. From media and emerging research, we know that parents have made a huge variety of arrangements for children during this period, which have been affected by the type of child care (or after school care) arrangements they had pre-pandemic, the availability of family close by, services offered by community organizations, etc. With our available data, we do not have the ability to consider all the relevant factors necessary to make predictions about which women were likely to exit the labor force.

- *In the paragraph beginning on line 433, there’s a highly cited framework called the NIOSH Hierarchy of Controls that refers to different strategies for risk reduction/mitigation. Suggest citing it here and using it as an organizing framework for this paragraph/section.*

This part of the discussion section is now rewritten and refers both to the HoC and to a thoughtful article applying this framework to COVID-19 hazard mitigation.

- *Finally, any analysis that looks at racial disparities should explicitly discuss systemic racism as an upstream driver of the distribution of race/ethnicity by occupations.*

Concerns about systemic racism and its consequences for workers’ location in the employment hierarchy and for health led us to undertake this research. Our research team has a long track record of research on racism, discrimination, and social inequality and health outcomes. However, in this paper, in order to complete and publish the research while it still can be useful to readers, we decided to focus on *the effects* of the occupational distribution of workers by race and ethnicity *on exposure* rather than on the *causes* of the occupational distribution of workers by race and ethnicity itself.

While we note the role of racism in occupational segregation, providing an adequate treatment of the social processes, including systemic racism, which have led to current occupational disparities by race and ethnicity would require a considerable lengthening of the paper and additional empirical analyses that would delay the availability of these results and greatly enlarge the scope of the paper.

- *Overall, this manuscript is very much in need of a coauthor or collaborator with expertise in occupational epidemiology, especially when using a job exposure matrix such as onet. There are clear gaps in knowledge (use of o*net, hierarchy of controls, intersection of occupation and industry classifications, testing for precision and accuracy of the assessed exposure) that should be addressed in a paper that explicitly addresses occupational exposure burden. I also suggest adding a social epidemiology coauthor or collaborator with experience in applying health disparities frameworks.*

The authors of this paper comprise a multidisciplinary research team whose members include epidemiologists and social scientists with an extensive track record of research on health disparities by race, ethnicity, and immigration status and the social causes of occupational attainment and social position. We also have considerable experience with analyses of O*NET, occupational and industry classifications, and occupational exposures. As noted above, this paper is intentionally sharply focused because, given our experience and background, we believe that it is essential to provide solid empirical results on the extreme disadvantage in terms of exposure to COVID-19 faced by many frontline workers during the pandemic, particularly among Black, Latino, Native American, and some other groups of workers.
