# Supplementary material for: Racial and ethnic differentials in COVID-19-related job exposures by occupational standing in the US
Source: PLoS One. 2021 Sep 1;16(9):e0256085. doi: 10.1371/journal.pone.0256085 (PMC8409606; doi:10.1371/journal.pone.0256085)
Supplement: Attachment — Submitted filename: Response to Reviewers.docx [file pone.0256085.s005.docx]

We thank the reviewers for their helpful comments. Below we provide responses to each of the reviewer’s comments. The reviewer’s comments are presented in italics.

**Reviewer 3:**

*The authors have addressed the majority of the comments/aspects raised by the reviewers. However, even if both reviewers raised concerns about a limitations section, the authors did not incorporate it. Although, authors included some sentences throughout the discussion about the concerns mentioned and the manuscript has been greatly improved, there are some issues that remain and are worthy of clarification. For instance, the issues of confounding and heterogeneity raised still hold. For instance, the response indicates that the study is only descriptive. However, this has not been stated anywhere in the text and the objective of "extending previous work on racial/ethnic differences in potential work-related exposure to COVID-19 by examining 12 racial/ethnic groups and by considering five indicators of potential risk exposure" including to "examine potential risk of exposure separately by occupational standing (OS)," may not be understood as only descriptive. Given the use of "control for OS", as stated by the authors in their answer. If the authors did not intended the estimation a causal effect and association, nor the interpretation as such, this could be explicitly presented in the abstract, objective/methods and discussion section of the manuscript as well, so the interpretations could be accordingly made.*

As noted previously, we did not intend this paper to provide a causal analysis. As suggested by the reviewer, we now explicitly state that the analysis is descriptive. We have added wording to the Abstract, Introduction, Analytic Strategy and Discussion sections to make this very clear. For example, in the Discussion we now say the following: “The analysis is descriptive – portraying potential workplace risks by race, ethnicity and gender – rather than an investigation of the root causes of these patterns.”

**Reviewer #4:**

*Thanks to the authors for the improvements to the paper, both minor and major.
I have just a few lingering concerns after which adopted, I will consider the paper to be publication ready.
A few places in your comments to reviewer you emphasize that O*NET covers all occupations, and that the ACS survey captures all occupations. I think the fallacy you are falling into is the assumption that the ~700 SOC codes covers all workers in the United States which is just not the case (you do acknowledge that military is not included in O*NET). Given the way O*NET is collected (where they reach out to workers/employers/experts about each of the occupations covered by SOCs), many workers are left out of the data collection—for example, Uber drivers wouldn’t be surveyed by O*NET, or a delivery worker for Amazon, a temporary worker for any organization, or a domestic worker being paid under the table, or even a general contractor, or a migrant agricultural worker. These workers may be surveyed by ACS, but their reported occupation is being matched to a census occupation code that isn’t very precise, and then you are further matching to O*NET data that was generated to represent a different population of workers (i.e. only those in more traditional employer-employee relationships covered by a SOC code). Employee respondents to O*NET are found through their employer. The authors acknowledge there is inner-occupation variability, but “there is not much we can do about it.” I am only asking for you to acknowledge these limitations—(1)collapsing all the diverse occupations self-reported in the ACS data to 400 occupations induces inner-occupation variability, and (2) in matching ACS to O*NET data, you are comparing two different populations (ACS is general US cross section, O*NET is a very specific set of occupations marked by a typical employee-employer relationship and is only available in English and Spanish) that have different distributions of occupational variability.
Neither of these limitations negate your work, but it has to be mentioned that O*NET does not cover all workers and indeed many of the workers it does not cover may be more “frontline” or at increased risk (and also could be more likely to be workers of color). By not acknowledging these “missing” workers that are continuously left out of BLS data collection it is further perpetuating their decreased standing and lack of importance and necessity to our economy.
Moreover, the lack of data from O*NET on the demographics of respondents makes it hard to ensure the sample is representative of the American workforce, though most assume that it is.
I would suggest reviewing this paper if you haven’t already:* [*https://onlinelibrary.wiley.com/doi/full/10.1002/ajim.20846?casa_token=MnvFp1NvrwUAAAAA%3ACffACaImtyoCu67O96UX_SBe49mfnSxZWlzrI8Rzkr9xmbeuL0uv3kVWI16Xb8geBNN2JnpXV_9n1Jg*](https://onlinelibrary.wiley.com/doi/full/10.1002/ajim.20846?casa_token=MnvFp1NvrwUAAAAA%3ACffACaImtyoCu67O96UX_SBe49mfnSxZWlzrI8Rzkr9xmbeuL0uv3kVWI16Xb8geBNN2JnpXV_9n1Jg) *Thank you for helping me understand the use of 1 year v. 5 year ACS. I agree that in this analysis the currency of the data is preferable to the precision of the data.*

We have changed the text in several places in the Discussion to make these points. We acknowledge that the ACS classification scheme has resulted in the combining of O*NET occupations thereby increasing intra-occupation variability. In a subsequent paragraph, we discuss the likely consequence of O*NET surveys being restricted to formal workers – i.e., underestimation of the extent of racial and ethnic differences in workplace exposures to COVID-19. We acknowledge that we are probably capturing substantial numbers of informal workers in the ACS data.
